# Tanshinone IIA: A Review of its Anticancer Effects

**DOI:** 10.3389/fphar.2020.611087

**Published:** 2021-01-14

**Authors:** Zhong‐ying Fang, Miao Zhang, Jia-ning Liu, Xue Zhao, Yong-qing Zhang, Lei Fang

**Affiliations:** ^1^ School of Biological Sciences and Technology, University of Jinan, Jinan, China; ^2^ School of Pharmacy, Shandong University of Traditional Chinese Medicine, Jinan, China

**Keywords:** *Salvia miltiorrhiza*, tanshinone IIA, anticancer, mechanism, traditional Chinese medicine

## Abstract

Tanshinone IIA (Tan IIA) is a pharmacologically lipophilic active constituent isolated from the roots and rhizomes of the Chinese medicinal herb *Salvia miltiorrhiza* Bunge (Danshen). Tan IIA is currently used in China and other neighboring countries to treat patients with cardiovascular system, diabetes, apoplexy, arthritis, sepsis, and other diseases. Recently, it was reported that tan IIA could have a wide range of antitumor effects on several human tumor cell lines, but the research of the mechanism of tan IIA is relatively scattered in cancer. This review aimed to summarize the recent advances in the anticancer effects of tan IIA and to provide a novel perspective on clinical use of tan IIA.

## Introduction

**FIGURE 1:**
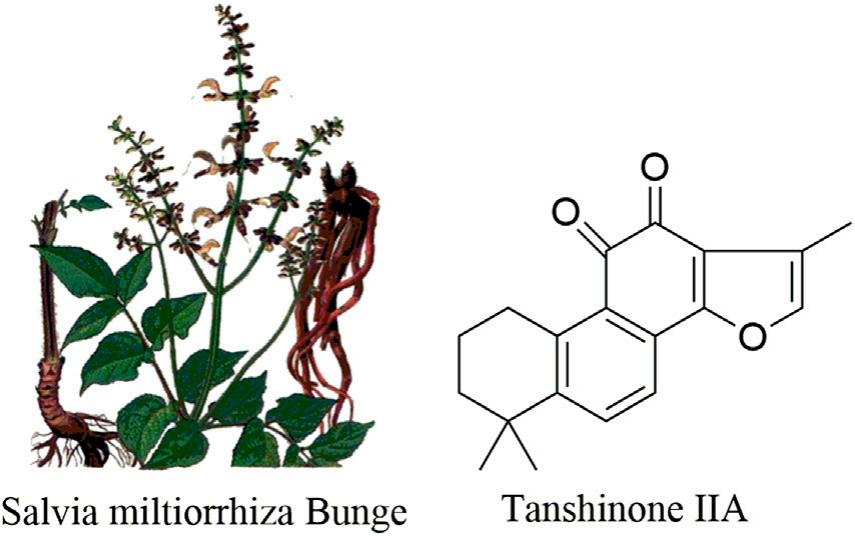
The plant of *Salvia miltiorrhiza* Bunge and the chemical structures of tanshinone IIA.

*Salviae miltiorrhiza* (Danshen) is the dried root and rhizome of *Salvia miltiorrhiza* Bge (Lamiaceae), which is a traditional Chinese medicine herb (Figure 1) (Tseng et al., 2014). It is mainly distributed in Anhui, Shanxi, Hebei, Sichuan, Shandong, Jiangsu, and other provinces in China and considered to have the action of relieving pain, activating blood circulation and removing blood stasis, clearing the heart and removing annoyance, cooling blood, and eliminating carbuncle, according to the mechanism of traditional Chinese medicine (TCM) (Li et al., 2016a; Zhou et al., 2019). There are two main active ingredients in *S. miltiorrhiza*. One is the hydrophilic component, which belongs to water-soluble substances, such as tanshinol, and the other is the lipophilic component, which belongs to fat soluble substances, such as tanshinone I and tan IIA (Kwak et al., 2008; Shang et al., 2012; Lin et al., 2019).

Tan IIA (C_19_H_18_O_3_, 14,16-epoxy-20-nor-5(10),6,8,13,15-abietapentaene-11,12- dione) (Wei et al., 2012), a natural diterpene quinone in *S. miltiorrhiza*, possesses miscellaneous biological activities such as anti-inflammatory (Dong et al., 2009; Li et al., 2015a; Fan et al., 2016), antiviral (Xiao et al., 2013; Zhang et al., 2014a), antioxidant (Gong et al., 2019), neuron-protective (Xia et al., 2005; Weng et al., 2018), antiatherosclerotic (Chang et al., 2014a; Tan et al., 2019), antiallergic (Li et al., 2018a; Heo and Im, 2019), anticonvulsant (Olivia et al., 2013), antifatigue (Lin et al., 2017), anti-Alzheimer’s disease (Jiang et al., 2014; Li et al., 2015b), and antiangiogenic activities (Fan et al., 2011), reducing organ damage (Ma et al., 2018a), and protection from angina pectoris and myocardial infarction (Lv et al., 2018) (its structure is shown in Figure 1). Newly, it was said that tan IIA could have a wide range of antitumor effects in multiple human tumor cell lines by inhibiting tumor growth, inducing apoptosis, regulating cell cycle, regulating signaling pathways, and reversing the multidrug resistance in various human tumor cells (Kim et al., 2015). However, because the mechanism of tan IIA cell is relatively scattered in cancer, this paper provides the research progress of the antitumor effect of tan IIA on leukemia, lung cancer, hepatocellular carcinoma, gastric carcinoma, colorectal cancer, glioma, osteosarcoma, cervical cancer, ovarian cancer, breast cancer, and prostate cancer, and its antitumor mechanism was also discussed.

## Antitumor Effects

Tan IIA could exhibit antitumor activity in many cancer cells such as leukemia, lung cancer, hepatocellular carcinoma, gastric carcinoma, colorectal cancer, glioma, osteosarcoma, cervical cancer, ovarian cancer, breast cancer, and prostate cancer. Tan IIA could induce autophagy and apoptosis and inhibit cell growth and migration, by activating AMPK and inhibiting PI3K/Akt/mTOR signaling pathway, and so on.

### Leukemia

Leukemia, including chronic myeloid leukemia (CML), acute myeloid leukemia (AML), and acute promyelocytic leukemia (APL), is one of the blood or bone marrow cancers. Some viruses, petrochemical products, ionizing radiation, and alkylating chemotherapy drugs are considered as major reasons of leukemia (Liu et al., 2012a). Around 100 million children and adults worldwide suffer from some forms of leukemia every year. At present, the treatment of leukemia remains a top research priority. In recent years, TCM has attracted wide attention as a clinical alternative to the treatment of leukemia because of its anti-inflammatory, antivirus, antioxidation, antitumor, apoptosis inducing effect (Boon and Wong, 2004). Among them, Tan IIA played an important role.

CML is a myeloproliferative disease. The translocation of chromosomes 9 and 22 leads to the clonal inflation of transformed hemopoietic stem cells, which may lead to resistance in tumor treatment (Yun et al., 2013a). Yun, et al. observed that tan IIA induced mitochondria dependent apoptosis through excitation of JNK in KBM 5 leukemia cells (Yun et al., 2013a). Tan IIA could raise sub-G1 apoptotic portion, activate Caspases 9 and 3, release cytochrome *c* from mitochondria into cytoplasm, and downregulate Survivin, Bcl-2, Bcl-xL, and c-IAP2 (Yun et al., 2013a). Then, they also discovered that tan IIA could induce autophagy via AMPK and ERK and restraint of mTOR and p70 S6K (Yun et al., 2013b).

AML is characterized by unlimited proliferation of myeloid cells (Liu et al., 2012a), with five-year mortality rate of more than 70% (Zhang et al., 2019). Therefore, we need to find more effective therapeutic strategies to treat AML. Zhang et al. revealed that tan IIA may induce apoptosis and autophagy in U937 cells via inhibiting PI3K/Akt/mTOR signaling pathway (Zhang et al., 2019). Tan IIA induced apoptosis in U937 cells via upregulating the levels of active Caspase 3 and Bax and downregulating Bcl-2. In addition, tan IIA inhibited the capacity of migration and invasion in U937 cells. Liu et al. discovered that tan IIA activated P × R (Pregnane × receptor), which inhibited nuclear factor-κB (NF-κB) activity, leading to significantly downregulating the expression of CCL2 by about ten times (Liu et al., 2012a).

APL is a seldom seen disease accounting for about 10% of AML. Zhang et al. indicated that C/EBPβ and CHOP participate in tan IIA induced variation and apoptosis of APL cells (Zhang et al., 2010). Tan IIA may upregulate C/EBPβ and CHOP; the C/EBPβ was very important (Zhang et al., 2010). Liu et al. suggested that tan IIA could induce apoptosis by excitation of Caspase 3, downregulation and upregulation of Bcl-2 and Bax, respectively, and the disruption of mitochondrial membrane potential (Liu et al., 2006). Moreover, the treatment by tan IIA may weaken adhesion and invasion of NB4 cells through the extracellular matrix (ECM). Yoon et al. demonstrated that induced apoptosis by tan IIA was accompanied by the PARP specific proteolytic cleavage and Caspase 3 activation (Yoon et al., 2000).

Liu et al. indicated that tan IIA has available antiproliferation effect on THP-1 cells by apoptosis; it is basically related to the destruction of Δψm (the mitochondrial membrane potential), activation of Caspase 3, and downregulation and upregulation of Survivin and Bax, respectively (Liu et al., 2009).

**FIGURE 2:**
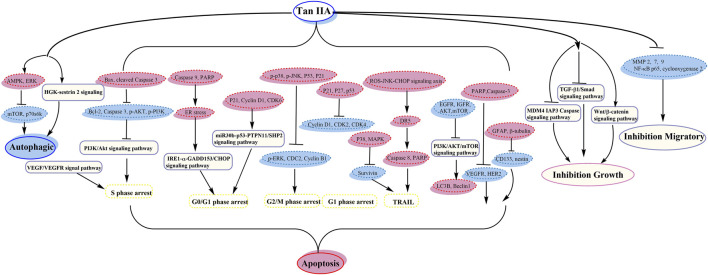
The anticancer pathway of tanshinone IIA.

Guo et al. demonstrated that nutlin-3 and tan IIA meaningfully potentiated the apoptotic effect of imatinib by downregulating AKT/mTOR pathway (Guo et al., 2017). Next year, they showed that the association of nutlin-3 and tan IIA may synergistically induce apoptosis, cytotoxicity, cell cycle arrest, and autophagy; thus the antileukemia effect was through effective activation of p53, inhibition of the AKT/mTOR pathway, and activation of the RAF/MEK pathway (Guo et al., 2018) (its anticancer pathway is shown in Figure 2).

### Lung Cancer

Lung cancer is a usual respiratory harmful tumor in the clinic; its morbidity is increasing with the change of modern circumstance and lifestyle (Li et al., 2016b). It has become the main cause of tumor related death in Taiwan and Western countries and has been one of the diseases which can strictly threaten the human life and fitness (Zhang et al., 2014b). With the consecutive ripeness of science and technology, the curative effect by pharmacon of lung cancer is greatly modified, but lung cancer remains fatal, widespread, and expensive to patients and society (Zhang et al., 2016). Therefore, how to efficiently treat lung cancer has been the focus of clinical investigation.

Xie et al. showed that tan IIA could restrain cell proliferation, induce apoptosis, and arrest cell cycle at the S phase (Xie et al., 2015). It may block VEGF/VEGFR signal pathway, cause cell cycle arrest, and indirectly inhibit downstream signal pathway and then upregulate the expression of apoptosis genes, downregulate antiapoptosis genes, then inhibit the development, and promote the apoptosis of tumor cells (Xie et al., 2015). Zhang et al. showed that tan IIA may induce cytochrome *c*-mediated caspase cascade apoptosis via the JNK pathway (Zhang et al., 2014b). It induced apoptosis through cytochrome *c* release from mitochondria and Bax migration to mitochondria (Zhang et al., 2014b). Wang et al. confirmed that the mechanism of tan IIA inhibiting cell proliferation and epithelial-mesenchymal transition (EMT) might be through the TGF-β1/Smad signaling pathway (Wang et al., 2018). Cheng et al. detected that tan IIA could inhibit H146 cells by upregulating Bax/Bcl-2 and diminishing mitochondrial membrane potential (Cheng and Su, 2010). The inhibition of tan IIA might be through endoplasmic reticulum (ER) stress caused through the release of Ca2^+^ and the increased expression of GADD153 protein. It induced the increase of Bax/Bcl-2 and Caspase 3 and the decrease in matrix metalloproteinases (MMP), leading to the suppression of the proliferation in H146 cells (Cheng and Su, 2010). Kim et al. have proven that tan IIA induced TRAIL sensitization of lung cancer cells by selective ER stress induction (Kim et al., 2016). So, tan IIA may induce apoptosis of TRAIL via upregulating DR5 and downregulating Survivin via selective activation of PERK/ATF4 and inhibition of STAT3, respectively.

Chiu et al. showed that tan IIA induced apoptosis by the abduction of ROS and diminishing the mitochondrial membrane potential in A549 cells (Chiu and Su, 2010). Tan IIA might decrease the expression of Bcl-2 and increase Bax, p53, and Cyto-c and may work via the abduction of ROS and a higher scale of Bax/Bcl-2. Liu et al. suggested that the apoptosis pathway by NQO1-activated and p53-independent mechanism determines the antitumor function of tan IIA against non-small cell lung cancer (Liu et al., 2012b). Tan IIA may activate ROS detonated, p53-independent, and caspase-dependent mitochondria apoptotic mechanism by increased Bax/Bcl-xL, disruption of mitochondrial membrane potential, release of cytochrome *c*, and caspase excitation and PARP-1 cleavage (Liu et al., 2012b).

Zu et al. indicated that tan IIA could inhibit the activity of p53 deficient H1299 cell practicability via the MDM4-IAP3-caspase signaling pathway and increase sensitivity to doxorubicin (DOX) (Zu et al., 2018). Liao et al. demonstrated that tan IIA together with cisplatin restrains non‐small cell lung cancer by downregulating the phosphatidylinositol 3-kinase/Akt signaling pathway (Liao et al., 2019). It could destroy migration and invasion, prevent the cell cycle in the S phase, and induce apoptosis (Liao et al., 2019). The expression of cleaved Caspase 3 and Bax was upregulated; nevertheless the expression of Caspase 3, *p*-PI3K, *p*-Akt, and Bcl-2 proteins was downregulated (Liao et al., 2019). Li et al. showed that tan IIA together with cyclophosphamide (CTX) could downregulate and upregulate Bcl-2 and Bax, respectively, inhibit the neovascularization of cancer organizations, and raise the immunological action, with a remarkable antitumor activity (Li et al., 2016b) (its anticancer pathway is shown in Figure 2).

### Hepatocellular Carcinoma

Hepatocellular carcinoma (HCC) is a common hepatic malignancy in the world and the occurrence rate of liver cancer has been sustained to increase over the last few decades (Chiu et al., 2018). The intricacy of the molecular etiopathogenesis and drug resistance of HCC brings great impediments in cure (Ren et al., 2017). Therefore, tremendous efforts have been devoted to developing effective antitumor drugs with less side effects. The ingredients isolated from traditional medicinal plants have attracted a wide range of interest (Ma et al., 2013; Chang et al., 2014b).

Dai et al. indicated that tan IIA showed anticancer effect on BEL 7402 cells through apoptosis and G0/G1 arrest (Dai et al., 2011). It induced apoptosis through excitation of calcium dependent apoptosis signaling pathways and upregulation of MT 1A (Dai et al., 2011). Lin et al. indicated that tan IIA contemporaneously induced both Nec-1 restraint and FLIPS regulation reconciled apoptosis/necroptosis in HepG2 cells (Lin et al., 2016). Chien et al. showed that tan IIA may inhibit J5 cell growth through increasing and decreasing Caspase 3 and CD31, respectively (Chien et al., 2012). Jeon et al. revealed that direct suppression of cytochrome P450 2J2 by tan IIA induced apoptosis (Jeon et al., 2015). The CYP2J2 inhibits carcinoma cell apoptosis via upregulation and downregulation of Bcl-2 and Bax, respectively (Jeon et al., 2015). Tan IIA has been said to produce cytotoxicity by apoptosis without generating mutations in the GSH/GSSG ratio (Lee et al., 2008). Wang et al. found that tan IIA could restrain the raised metastasis induced by PR of hepatocellular carcinoma and drag on survival in part through VEGFR1/PDGFR-related vascular normalization (Wang et al., 2012). It immediately heightened tube formation of TECs, associated with VEGFR1/PDGFR upregulation. Ren et al. found that the multiple mechanisms involved in tan IIA induced death formed from miR30b-p53 pathway and PTPN11/SHP2 pathway (Ren et al., 2017). The transsituation of p53 may be the original signal, and miR30b-p53-PTPN11/SHP2 may be a fresh signaling pathway concerned in tan IIA induced cell death (Ren et al., 2017). Tan IIA could change Bax/Bcl2, p21, Caspase 3, cyclin D1, and CDK6, and it induced apoptosis through arresting cell cycle at G1/G0 phase (Ren et al., 2017).

Chiu et al. detected that tan IIA and sorafenib or SC-1 have collaborative cytotoxicity (Chiu et al., 2018). Tan IIA could restrain HCC proliferation via downregulation of pSTAT3 induced by sorafenib/SC-1. Chang et al. investigated that combination of tan IIA and trans-resveratrol (Resv) could raise the effect of apoptosis, sub-G1 arrest, and DNA fragmentation (Chang et al., 2014b). Tan IIA blocked the cells at sub-G1 phase, while Resv induced S and G2/M phase arrest, and tan IIA provoked a marked feature of thanatosis, while Resv mainly induced apoptosis (Chang et al., 2014b). Kan et al. suggested the accession of DOX cytotoxicity by tan IIA (Kan et al., 2014) (its anticancer pathway is shown in Figure 2).

### Gastric Carcinoma

Gastric carcinoma is an ordinary malignancy around the world and is a great threat to public health worldwide, with the second central consideration of tumor related death (Chen et al., 2012). It has the characteristics of high incidence rate, invasion, and incidence rate and poor prognosis (Dong et al., 2008). The familiar treatment of gastric cancer is gastrectomy together with chemotherapy and chemotherapy which has multidrug resistance and cytotoxicity to regular cells (Chen et al., 2012). Heretofore, many methods have been applied by the scientists to surmount the drug resistance in cancer (Xu et al., 2018). The effective chemical components from herbal medicine may contribute to improving the therapeutic effect of gastric cancer patients (Zhang et al., 2018a). Natural products are the leading compounds in the development of anticancer drugs and show versatile anticancer actions and have attracted more and more attention (Xu et al., 2018). Therefore, it is an emergency work in clinical practice to find a new treatment (Dong et al., 2008).

Dong et al. indicated that tan IIA could induce apoptosis of MKN 45 cells, and it may happen in G2/M phase; the possible molecular mechanisms are downregulation and upregulation of Bcl-2 and p53, respectively (Dong et al., 2008). Yu et al. found that tan IIA suppresses SGC 7901 cell proliferation and transplantation by downregulation of FOXM1 (Yu et al., 2017). Chen et al. indicated that tan IIA may cause cycle arrest in the G2/M phase and produce intrinsic apoptotic signaling pathway (Chen et al., 2012). Zhang et al. revealed that tan IIA inhibited cell proliferation and tumor growth via downregulating STAT3 (Zhang et al., 2018a). The treatment of tan IIA might induce apoptosis; it may increase cleaved Caspase 3 and decrease Bcl-2 (Zhang et al., 2018a).

Su et al. showed that tan IIA could inhibit AGS cell germination via decreasing Mcl-1, TCTP, BiP, and Bcl-xL and increasing Bax and CHOP (Su, 2014a). The same year, they suggested that tan IIA suppressed AGS cells by increasing the expression of p53, *p*-p38, and *p*-JNK and reducing the expression of *p*-ERK, CDC2, and cyclin B1 (Su, 2014b). One of the molecular mechanisms might be to increase *p*-p38 and *p*-JNK and decrease *p*-ERK to induce the activation of p53 and increase the expression of p21 to downregulate CDC2 and cyclin B1, which then induces G2/M phase arrest (Su, 2014b). Another way may be to improve the expression of TNF-*α*, FAS, and Caspases 3 and 8 to induce apoptosis (Su, 2014b). Second year, they reported that tan IIA decreased the migratory ability via decreasing the expression of MMP-2, MMP-7, MMP-9, NF-κB-p65, and cyclooxygenase-2 (Su, 2015). Third year, they documented that tan IIA may restrain AGS cells by decreasing EGFR, IGFR, AKT, and mTOR and blocking the PI3K/Akt/mTOR pathway (Su and Chiu, 2016). Then, they showed that tan IIA may induce AGS cells apoptosis by decreasing VEGFR and HER2, blocking the Ras/Raf/MEK/ERK pathway, and inducing the excitation of PARP and Caspase 3 (Su, 2018).

Xu et al. demonstrated that tan IIA might raise the antitumor effect of DOX in drug-resistant gastric cancer cells, by inhibiting MRP1 function, enhancing cell cycle arrest, and increasing apoptosis and autophagy (Xu et al., 2018) (its anticancer pathway is shown in Figure 2).

### Colorectal Cancer

Colorectal cancer is the third most ordinary type of human tumor because it is closely related to a range of factors according to the World Health Organization (Ma et al., 2018b). With the development of medical technology, great advancement has been made in the diagnosis and treatment of this disease, but recent chemotherapeutic plans are ungratified and the recurrence and death rate of colon cancer are still high (Su et al., 2008). Accordingly, the development of new treatment methods is particularly important. Among others, the phylactic and remedial capabilities of natural products of restraining or overturning cells associated with cancer conception, advancement, and succession are accepting much attention (Su et al., 2008).

Su et al. investigated that tan IIA may induce apoptosis via downregulating ErbB-2 (erythroblastosis oncogene B; HER-2/neu) and upregulating TNF-*α* in colon cancer cells (Su and Lin, 2008a). And then, they suggested that tan IIA triggered apoptosis by activating both inherent pathways concerning mitochondrial release of cytochrome c and outside pathways concerning excitation of Fas-caspase cascades (Su et al., 2008). Finally, they supported that tan IIA may build up the effectiveness of 5-FU in a colon cancer nude SCID mouse model by downregulating expression of NF-κB-p65, VEGF, P-gp, MMP-7, and LC3-II (Su, 2012).

Ma et al. showed that tan IIA may restrain COX-2 and activate Wnt/β-catenin signaling pathway, downregulate VEGF, and result in inhibition of colon cancer cells (Ma et al., 2018b). COX-2 is an important rate-limiting enzyme in the synthesis of prostaglandins; it could produce prostaglandin PEG_2_ after metabolism, which could increase the proliferation of cells and reduce the cell death. Tan IIA could also cause a β-catenin decrease, and Wnt/β-catenin signaling pathway is a highly conserved cell signaling system (Ma et al., 2018b).

Tu et al. supported that tan IIA might ameliorate inflammatory microenvironment through inhibiting of microRNA-155 and repressing the proliferation of Hct116 and Ht29 cells (Tu et al., 2012). Bai et al. revealed that tan IIA may induce cell death and enhance sensitizing to 5-FU cure by restraining excitation of NF-κB (Bai et al., 2016) (its anticancer pathway is shown in Figure 2).

### Glioma

Glioma is the most common primary central nervous system cancer, which has the characteristics of high invasiveness, high recurrence rate, and poor prognosis (Ding et al., 2017). So far, the treatment options of malignant glioma patients were still limited, which has an important impact on human health (Wang et al., 2007). Due to the limitations of current treatment methods, it is necessary to develop novel therapeutic strategies according to the specific biological characteristics of this tumor (Wang et al., 2007). It is very considerable to find more valid therapy to further antitumor effect and drag on the survival of patients (Ding et al., 2017).

Ding et al. verified that tan IIA could induce apoptosis and autophagy and raise LC3B and Beclin 1 and play an antitumor role by restraining the PI3K/Akt/mTOR pathway (Ding et al., 2017). It could decrease *p*-PI3K, *p*-Akt, and Bcl-2, increase Bax, restrain viability of cells, and facilitate apoptosis. Tang et al. showed that tan IIA efficiently inhibited the STAT3 pathway and downregulated Bcl-xL and cyclin D1 which were targets of STAT3, induced apoptosis, and inhibited tumor cell growth in C6 glioma cells (Tang et al., 2010). Wang et al. suggested that the cells treated by tan IIA showed astrocyte or nerve fiber-like modalism, and tan IIA increased GFAP mRNA, decreased nestin mRNA meaningfully, increased apoptotic cells expressively, increased cells in G0/G1 phase and decreased cells in S phase, and increased expression of ADPRTL1 and CYP1A1 mRNA (Wang et al., 2007).

Yang et al. suggested that tan IIA might increase variation and neural lineage flags including GFAP and β-tubulin, decrease glioma stem cells (GSCs) flags including CD133 and nestin, and then induce GSC apoptosis (Yang et al., 2014). Tan IIA inhibited the growth by interrupting IL6/STAT3 signaling pathways, not only reducing expression of IL6, but also reducing activated STAT3 (Yang et al., 2014) (its anticancer pathway is shown in Figure 2).

### Osteosarcoma

Osteosarcoma is a highly invasive tumor, which is the most common elementary malignant cancer in adolescents and young people, and it mainly occurs in the areas of positive bone growth and renovation (Yen et al., 2018). The characteristics of osteosarcoma are high metastatic expanding liability, poor prognosis, and low patient survival rate (Ma et al., 2016). Present treatment strategies include chemotherapy and aggressive surgical resection, but the five-year survival rate remains 5–20% (Ma et al., 2016). In recent years, more and more evidence has shown that TCM could apply potential drugs to prevent or treat various cancers (Zhang et al., 2012a). Therefore, it is of great practical significance and urgency to develop new plan to restrain recrudescent and intractable osteosarcoma and research the mechanism of antitumor effect (Huang et al., 2017).

Ma et al. confirmed that interaction between Beclin-1-dependent autophagy and caspase-dependent apoptosis is induced by tan IIA. ROS play a central part in adjusting the cytotoxicity of tan IIA (Ma et al., 2016). Yen et al. discovered that HGK-sestrin 2 signaling-mediated autophagy is advantageous to anticancer effectiveness of tan IIA (Yen et al., 2018). They defined the activation of HGK/SAPK/JNK1/Jun kinase pathways in upregulating transcription of SESN2, in which tan IIA invited HGK/JNK1-dependent Jun excitation and gave rise to increasing Jun recruitment to AP-1-binding site in the SESN2 promoter region (Yen et al., 2018). Zhang et al. showed that tan IIA induced apoptosis and inhibited the diffusion, transference, and aggression in MG-63 cells (Zhang et al., 2012a). It could inhibit mRNA, MMP-2, and MMP-9, restrain cell aggression though Matrigel, and reduce MG-63 migration activity (Zhang et al., 2012a). Huang et al. demonstrated that tan IIA could induce apoptosis through inherent pathways and result in mitochondrial lesion and suppress vasculogenesis (Huang et al., 2017). The balance of mitochondrial fission/fusion and the adjustment of cancer vasculogenesis were also relevant in the new antitumor effect of tan IIA (Huang et al., 2017) (its anticancer pathway is shown in Figure 2).

### Cervical Cancer

Cervical cancer (CC) is one of the most ordinary gynecological harmful cancers that severely threaten women health and is one of the major causes of female death all over the world (Qin et al., 2018). High risk human papilloma virus (HPV) infection plays a crucial role in the multifactor etiology (Munagala et al., 2015). New therapeutic drugs and effective antitumor cure hinge on developments in investigation. In recent years, the investigation has shown that certain TCM has showed antiviral and tumor apoptosis properties (Pan et al., 2010).

Munagala et al. suggested that tan IIA strongly restrained diffusion of C33a, CaSki, HeLa, and SiHa cells (Munagala et al., 2015). Tan IIA was found to downregulate HPV E6 and E7; regulate associated E6AP and E2F1; create S phase cell cycle arrest; attract accumulation of p53 and alter p53-dependent targets; modulate pRb; cause p53-mediated apoptosis by moderating Caspase 3, Bcl-2, Bax, and PARP cleavage in HPV positive CaSki cells. It could repress HPV E6 and E7 and then result in breeding of p53-dependent cancer allayer liveness resulting in growth inhibition (Munagala et al., 2015).

Zhou et al. concluded that tan IIA might prevent cancer cells in mitosis via disorganizing the mitotic spindle and after that trigger cells to enter apoptosis via the mitochondria dependent apoptotic pathway in HeLa cells (Zhou et al., 2008). It may selectively kill mitotic cells over interphase cells and destroy only the mitotic spindle during the M phase but not the microtubule structure in interphase cells (Zhou et al., 2008). Pan et al. demonstrated that it may firmly bind to the β-subunit of the microtubule protein, and it strongly inhibits the growth by interfering with the process of microtubule assembly, then resulting in G2/M phase arrest and sequent apoptosis (Pan et al., 2010).

Pan et al. evidenced that tan IIA could reveal tough growth prohibitive effect on CaSki cells by accelerating caspase cascades with concomitant upregulation of the phosphorylation of p38 and JNK signaling (Pan et al., 2013). Qin et al. showed that tan IIA restrained the migration and invasion of cervix carcinoma stem cells via inhibiting YAP transcriptional activity (Qin et al., 2018) (its anticancer pathway is shown in Figure 2).

### Ovarian Cancer

Ovarian cancer is one of the most ordinary human malignancies and results in death from gynecological malignancies (Huang et al., 2015). Due to the lack of sensitive and specific early detection methods, the diagnosis of ovarian cancer is usually late, and the treatment plan is limited (Li et al., 2018b). So, the development of antiapoptotic TCM monomers has been the center of surveys in the remedy of cancer (Zhang et al., 2018b).

Zhang et al. showed that tan IIA could induce apoptosis via attenuation of PI3K/AKT/JNK signaling pathways (Zhang et al., 2018b). It meaningfully increased the apoptosis by cleavage excitation of Caspases 3, 8, and 9 (Zhang et al., 2018b). Huang et al. investigated that it induced arrest of cell cycle at the G2/M phase, decreased Bcl-2, increased Bax, promoted SKOV3 cell apoptosis, and inhibited cell proliferation and viability (Huang et al., 2015). Li et al. demonstrated that it may induce apoptosis in TOV-21G cells via direct upregulation of miR-205 and in turn downregulation of Survivin (Li et al., 2018b). Chang et al. confirmed that tan IIA enhanced tumor necrosis TRAIL-induced apoptosis by upregulating DR5 acceptor by the ROS-JNK-CHOP signaling axis (Chang et al., 2015). Lin et al. illustrated that tan IIA enhanced the effect of TRAIL by downregulating Survivin in ovarian carcinoma cells (Lin et al., 2015). The downregulation of Survivin induced via tan IIA requires p38 MAPK activation and is regulated by both transcription process and proteasome degradation.

Jiao et al. revealed that tan IIA has remarkable antiproliferative effect on COC1/DDP cells by generating apoptosis and downregulating cisplatin-resistance genes (Jiao and Wen, 2011). The apoptosis was mainly related to the reduction of Survivin, and lessened cisplatin resistance was invited by the reduction of ERCC1 and LRP (Jiao and Wen, 2011) (its anticancer pathway is shown in Figure 2).

### Breast Cancer

Breast cancer is one of the most ordinary malignancies and the main reason of cancer death for women all over the world (Yu et al., 2014). It has the characteristics of high recurrence rate, high metastasis rate, and high mortality (Lin et al., 2013). For the past few years, with the increasing incidence rate and mortality of breast cancer, more and more attention has been paid to research of chemotherapy and new anticancer drugs; it has prompted people to seek more prevention and treatment methods (Li et al., 2015c).

Yan et al. found that tan IIA inhibited BT 20 cells by increasing Caspase 12, GADD153, Caspase 3, phospho-JNK, phospho-p38, and Bax and decreasing Bcl-xL and phospho-ER (Yan et al., 2012). Its molecular mechanism might be by generating ER stress and the MAPK pathway (Yan et al., 2012). Wang et al. confirmed tan IIA inhibition of proliferation and apoptosis through upregulation (CDKN1A, ARHC, CYP1A1, CLU, and ADPRTL1) and downregulation (MAP3K1, CEACAM6 and MMP-7) of many genes containing cell cycle management, signal transduction, cell propagation, apoptosis, vasculogenesis, invasion, and metastasis of cancer cells (Wang et al., 2005). Lu et al. suggested that tan IIA proved a stable prohibitive effect on the diffusion of ER-positive and ER-negative breast cancer cells by reducing P53 and Bcl-2 (Lu et al., 2009). Su et al. showed that tan IIA restrained MDA-MB-231 cells via increased Bax/Bcl-xL (Su and Lin, 2008b). Tan IIA increased p21 and Caspase 8. p21 (WAF1 and Cip-1) has the feasibility to induce arrest of G1 and apoptosis. Then, they indicated that tan IIA restrained cells by reducing LC3-II, Erb-B2, and NF-κB-p65 (Su et al., 2012). Li et al. showed that tan IIA restrained the vasculogenesis and growth via the suppression of hypoxia-inducible factor 1*α* (HIF-1*α*) synthesis and VEGF, in which the mTOR/p70S6K/4E-BP1 signaling pathway was participating (Li et al., 2015c).

Lin et al. showed that tan IIA restrained breast cancer stem cells growth via attenuation of IL-6/STAT3/NF-κB signaling pathways (Lin et al., 2013). After tan IIA treatment, the expression of IL-6, NF-κB-p65, phospho-STAT3 (Tyr705), and STAT3 in nucleus and cyclin D1 were reduced meaningfully (Lin et al., 2013).

Fu et al. reported that it may ameliorate hypoxia induced iatrochemistry obstruction to DOX and EMT, which may be attributed to the downregulation of HIF-1*α* (Fu et al., 2014). Li et al. demonstrated that tan IIA might boost the susceptivity to DOX by restraining the PTEN/AKT pathway and downregulating efflux ABC transporters incorporating MRP1, BCRP, and P-gp (Li et al., 2019). Li et al. suggested that it enhanced the chemosensitivity to DOX via reducing MDR-related ABC transporters (Li and Lai, 2017). It could facilitate endocellular DOX cumulation of MCF-7 cell and increase the sensitivity to DOX (Li and Lai, 2017). Lin et al. showed that tan IIA may shorten the taxol resistance via inhibition of the tau expression in MCF‐7 cells (Lin et al., 2018) (its anticancer pathway is shown in Figure 2).

### Prostate Cancer

Prostate cancer is the most ordinary aggressive cancer and the second major consideration of death in men (Chiu et al., 2013). At present, the remedial formations of prostate cancer generally have drug resistance and high toxicity (Zhang et al., 2012b). Therefore, it is still the priority of prostate cancer investigation to find a more effective chemical prevention plan with the least side effects (Gong et al., 2011).

Won et al. observed that tan IIA may induce mitochondria dependent apoptosis by inhibiting PI3K/AKT survival pathway (Won et al., 2010). It may reduce PI3K, p85 subunit, and the phosphorylation of AKT and mTOR (Won et al., 2010). Tan IIA induced p53 excitation and mitochondrial lesion, resulting in Caspase 9/ 3 reconciled apoptosis (Won et al., 2010). Then, they demonstrated that it induced arrest of G1 through activation of p53 signaling and restraint of androgen receptor (AR) in LNCaP prostate cancer cells (Won et al., 2012). Tan IIA could induce arrest of cell cycle at G1 phase and reduce cyclin D1, CDK2, and CDK4 and activate the phosphorylation of p53 at Ser 15 residue and its downstream p21 and p27 (Won et al., 2012). Chiu et al. suggested that it restrained growth by reaction of ER stress (Chiu et al., 2013). It could arrest cell cycle G0/G1 and the underlying mechanism for apoptosis via the excitation of PARP, Caspases 9/3, and the reaction of ER stress through the IRE1-*α*-GADD153/CHOP pathway (Chiu et al., 2013).

Li et al. showed that the apoptosis and autophagy were dependent on the ROS abducted via tan IIA in PC-3 cells (Li et al., 2015d) (its anticancer pathway is shown in Figure 2).

## Conclusion

Recently, TCM has played more and more important roles in health conservation, the prevention and treatment of diseases, and plant drug detection. The use of TCM to prevent the occurrence and revolution of multiple malignant diseases has become an important choice for the cure of malignant disease. There has been great effort not only to develop new drugs, but also to conclude how the consisting ingredients exhibit their activities. *S. miltiorrhiza* has been widely used in eastern countries, especially in China, to treat miscellaneous diseases for its extraordinary pharmacological actions, including free radical scavenging, anticoagulation, and vasodilatation.

Tan IIA is an effective component in the extract of *S. miltiorrhiza* Bunge, which has been diffusely used in TCM exercise for more than thousand years to treat diverse diseases. It significantly induced apoptosis on a panel of cancer cells, such as leukemia, lung cancer, hepatocellular carcinoma, gastric carcinoma, colorectal cancer, glioma, osteosarcoma, cervical cancer, ovarian cancer, breast cancer, and prostate cancer.

Overall, Tan IIA has remarkable prohibitive effect on a variety of tumor cells and its possible mechanism involves regulating cell cycle, inhibiting cell diffusion, inducing apoptosis and differentiation, inhibiting tumor aggression and diversion, inhibiting angiogenesis and reversing tumor MDR, and so on. Tan IIA, as a sort of medicine possessing multiple pharmacological actions, has the characteristics of high efficiency, low toxicity, and natural source and possessed considerable potential value clinically. The combination of tan IIA and other clinical commonly chemotherapeutic drugs could enhance the therapeutic effect of chemotherapeutic drugs, which makes tan IIA have a good application prospect in tumor therapy and adjuvant therapy and also provides a new idea for various cancer treatment.

## Author Contributions

Z-YF collected the documentations and wrote the original manuscript; MZ, J-nL, and XZ classified the pharmacological literatures; Y-qZ and LF proposed amendments and modified the paper. All authors contributed to the article and approved the submitted version.

## Funding

This work was supported by the National Key Research and Development project (2017YFC1702702) and Shandong Provincial Technological Innovation and Guidance Project (2017YFC1702702).

## Conflict of Interest

The authors declare that the research was conducted in the absence of any commercial or financial relationships that could be construed as a potential conflict of interest.
